# Can the green credit guidelines effectively deter enterprise inefficient investment of innovation? -evidence from heavy polluting enterprises in China

**DOI:** 10.1371/journal.pone.0298097

**Published:** 2024-02-23

**Authors:** Chuanhua Song, Shengli Jiao, Zengjun Sun

**Affiliations:** 1 Graduate School, Namseoul University, Cheonan, South Korea; 2 Graduate School, Jeonju University, Jeonju, South Korea; Jiangsu University, CHINA

## Abstract

The green credit policy serves as a crucial instrument for achieving the dual objectives of optimal resource allocation and green development. It plays a pivotal role in curbing inefficient investments in innovation by enterprises. This research employs the PSM-DID method to effectively explore the practical effects of the green credit policy on the innovation inefficiency investments of heavily polluting enterprises in China. Examining the impact from the perspectives of environmental regulation and financial constraints, the study utilizes panel data from listed companies on the Shanghai and Shenzhen A-shares markets spanning from 2010 to 2020. The following conclusions are drawn: (1) Green credit policy has proven effective in inhibiting the inefficient investment in innovation by heavily polluting enterprises when compared to non-heavily polluting enterprises. (2) Moreover, this effect is more pronounced in state-owned enterprises and regions with less financial development. (3) Mechanism testing reveals that the green credit policy can discourage corporate over-investment by influencing financing constraints and can alleviate under-investment through commercial credit.

## 1. Introduction

With the acceleration of economic development and industrialization, the environmental problems worldwide have gradually affected ecological civilization and green development [1]. Environmental pollution is related to a highly polluting industrial structure, energy structure and transportation structure, especially in China, and heavy polluting enterprises consume a lot of resources and energy, thus aggravating environmental pollution [2]. As the world faces the challenge of environmental degradation, more and more firms are taking innovative measures to achieve the goal of sustainable economic growth, especially the heavy polluters who were widely believed to bear greater responsibility [3]. On the one hand, as the cost of regulation becomes higher, it is more challenging to pass on the cost of regulation to consumers, thus leading to increased demand for firm innovation [4]. On the other hand, restricting the use of polluting technologies or mandating the use of cleaner technologies will result in higher hidden emission costs for firms, thus stimulating firm innovation [5]. Heavy polluters urgently need to improve their production structures and processes through a series of innovative activities to meet the requirements of the government and the public [6].

However, the long periodicity of enterprise technology innovation, positive spillover effect and large investment amount make corporate innovation face high innovation risk and information asymmetry [7]. Especially the bank-dominated financial system in China makes enterprise innovation investment face more serious financial constraints [8]. On the one hand, enterprises, especially heavy polluters, face financing problems; on the other hand, environmental sustainability requires significant investments and banks are currently the main source of financing to support sustainable economic development and green investments [9]. To substantially solve environmental problems, it is necessary not only to rely on stronger end-of-pipe measures [10], but also to adopt a series of fiscal and financial instruments to change the incentive mechanism for resource allocation [11].

The Green Credit Guidelines (GCG), issued by the China Banking Regulatory Commission (CBRC) in 2012, institutionalized the binding of China’s green finance policy with corporate environmental performance. By providing more loans or preferential interest rates for environmentally friendly projects, the GCG aims to adjust the flow of funds from credit resources to eventually promote a green transformation and industrial upgrading [12]. The implementation of GCG will reduce the scale of debt financing for heavy polluters. Based on information asymmetry theory, banks tend to restrict loans to HPEs, especially long-term loans [13]. For one thing, under the condition of limited financial resources, investment in environmental management must have a “crowding-out effect” on productive investment in the short term; for another, the capital-intensive characteristics of the products are becoming more and more obvious, and the financing constraints of enterprises are more serious [14].

The impact of environmental regulation on enterprise innovation behavior is a top priority in current economic research. Recent studies have shown GCG can reduce environmental pollution through improving enterprise performance, motivating innovation and upgrading industrial structure. For example, Du et al. (2019) [14] investigated how informal environmental regulation, like public environmental appeal, exerts positive effect on innovation efficiency [15].

According to previous research findings, most of them have done studies that focus more on innovation outcomes [16–19], but rarely investigate the efficiency of innovation investment. Therefore, this paper investigates whether implementation of the Green Credit Guidelines (GCG) can inhibit the inefficient investment of innovation in heavy polluting enterprises (HPEs) in China and, if so, through which mechanisms [20].

This study contributes to the literature in the following ways. Firstly, in terms of contribution to the existing literature, this study explores the impact of GCG on innovation investment efficiency of HPEs from the micro perspective, which further enriches the innovation research within the field of sustainable development. Secondly, in terms of contributions to the research content, this paper has the following contributions: 1) it started from the end of innovation input, to verify the effect of GCG on inefficient investment of innovation of HPEs, while existing studies that focus on innovation outcomes. This not only is an effective supplement to the existing policies like GCG in the financial field and empirical research field, but also provides more favorable solutions for enterprises to better control innovation investment decisions and improve the effectiveness of innovation investment. 2) it discusses the mechanism of the impact of GCG on the efficiency of innovation investment in HPEs from two perspectives: SA index and commercial credit, analyzing the over-investment in innovation and under-investment in innovation, respectively.

## 2.Literature review

### 2.1. Green credit policy

The origin of green credit can be traced back to the "Comprehensive Environmental Response, Compensation and Liability Act (CERCLA)" passed by the United States in 1980. Then the Equator Principles (referred to as EPs) approved by the world’s major financial institutions at the London Conference in 2002 constitute an important milestone for the banking industry, which has established the minimum industry standards for the environment and society of international project financing for the first time and developed into an industry practice. In 2012, the China Banking Regulatory Commission (CBRC) published the Green Credit Guidelines, because the GCG practically can promote green development by tilting resources to environmental projects, through the economic approach to facilitating green industrial structure transformation and upgrading of the optimal impact [21]. Since 2015, China has deeply promoted the construction of ecological civilization and economic structural transformation. In 2016, the G20 Summit proposed to vigorously develop green credit. Until today, green credit policy has been of great interest to theoretical and practical research.

The GCG has made clear and specific arrangements for the green credit business of financial institutions to ensure that credit funds are invested in the green, low-carbon economy [10]. As a critical environmental regulatory tool, the role of GCG in environmental governance has received increasing attention in recent years. Some studies discuss the relationship between GCG and health, and sustainable development from the macro perspective, mostly related to the economic impacts, industrial structure impacts [22] and environmental impacts [23]. For instance, S. Zhang et al. (2021) [23] stated that the GCP improves environmental quality by influencing the behavior of “two high” enterprises and contributes to the mitigation of sulfur dioxide and wastewater emissions. Y. Hu et al. (2020) [22] explored the mechanism of how the GCG influences the industrial structure through capital and funding channels of enterprise, and proposed for the related stakeholders, promoting the transformation of industrial structure should be based on the local condition.

From the micro perspective, recent studies investigate the impact of GCG on enterprise innovation [16,17], resource allocation [19,24] and green transformation of HPEs. For example, Wen et al. (2021) [19] explored the impact of GCG on the upgrade of energy-intensive enterprises and further found GCG had reduced the allocation efficiency of bank credit within energy-intensive industries. Zhou et al. (2021) [24] used the triple difference method and verified the impact of GCG on credit resources and financing costs of enterprises. Tian et al. (2022) [25] stated that the GCG significantly promotes the green transformation of HPEs, through debt financing constraints and equity financing constraints in the short term.

The development of green credit, as one aspect of financial development, will alleviate uncertainties, improve the level of credit allocation and enhance the effectiveness of GCG, thus have a sustainable and stable impact on enterprise financing. By inducing banks to take the environmental information of enterprises into their lending decision, GCG will reduce the information asymmetry between banks and enterprises [26].

### 2.2 Factors affecting enterprise innovation

Previous papers have studied the driving factors for enterprise innovation, and divided them into external and internal factors. External factors contain environmental regulation [27,28], digital transformation [29], government subsidies, and green financial reform. For example, Liao (2018) [28] constructed a logical line of “policy-behavior-performance” to test the impact of environmental policy instruments and its combination on the enterprise’s environmental innovation. Borghesi et al. (2015) [27] analyze how the technological and organizational innovations have been influenced by policy and regulation levers in the EU from the sectoral perspective, by exploring the single and interaction effects of policies, especially emission trading. Shao et al. (2020) [5] summarized the impacts of environmental regulation on enterprise innovation from the perspectives of technological innovation, product innovation, system innovation, and ecological innovation. Wang and Sawur (2022) [30] confirmed that government subsidies and investments play a positive moderating role in the impact of environmental regulation on green enterprise innovation.

Internal factors emphasize shareholder pressure [31], insider management, corporate governance [32], corporate profitability [33], and manager characteristics [34]. From the internal view, F. Zhang & Zhu (2019) [31] found that stakeholder pressure through green dynamic capability influences green innovation, and finally influences firm performance. Rong et al. (2017) [35] found that the presence of institutional investors enhances firm innovation through mutual funds and its heterogeneous performance. Singh et al. (2021) [36] suggested that top management knowledge value and knowledge-creating practices influence open innovation, which, in turn, influences organizational performance.

There is no consensus among the researchers on the issue of how environmental regulatory policies affect firm innovation. Some scholars based their studies on the neoclassical economic theory that environmental regulation increases the environmental compliance costs of enterprises [37], thus inhibiting their green innovation, especially for HPEs. Other literature explored evidence to support Porter Hypothesis that firms will increase their R&D innovation intensity in the face of increased environmental regulation. Besides, recent studies have also found a U-shaped relation between environmental regulation and enterprise innovation [36,38].

Existing research on the measurement of firm innovation mostly adopts output outcomes like the number of green patent application data [39], questionnaire data, new product sales data, the number of patents acquired as the indicators of innovation [5] For example, Z. Zhang et al (2022) [3] stated that GCG improves the overall and incremental green innovations but impedes the radical green innovation of highly polluting enterprises with the framework of environmental policy, local government intervention and enterprise innovation.

In summary, most of the existing research results support the "Porter hypothesis", which also provides theoretical support for this study. GCG is a market-based regulation that reshapes the investment and financing mechanism of the environmental sector from the capital supply side outside the government sector. Its essence is an important extension and innovation of traditional environmental regulation. Enterprises that are more strongly affected by environmental regulations are more willing to carry out R&D innovation and have a larger amount of R&D expenditure. However, the implementation of a green credit policy will raise the financing threshold in the short term and directly aggravate the financing difficulty of enterprises to some extent. Therefore, compared with non-heavy polluting enterprises, when HPEs are faced with tighter credit regulation standards, they are more motivated to improve the quality of green development by improving innovation efficiency, to reach the new threshold of green credit.

### 2.3 Research on the influence of green credit on enterprise innovation

There is no consensus among the researchers on the issue of how environmental regulatory policies affect firm innovation. Some scholars based their studies on the neoclassical economic theory that environmental regulation increases the environmental compliance costs of enterprises (Gollop Boston et al., 1971) [37], thus inhibiting their green innovation, especially for HPEs. Other literature explored evidence to support Porter Hypothesis that firms will increase their R&D innovation intensity in the face of increased environmental regulation. Besides, recent studies have also found a U-shaped relation between environmental regulation and enterprise innovation.

Existing research on the measurement of firm innovation mostly adopts output outcomes like the number of green patent application data [38–40], questionnaire data, new product sales data, the number of patents acquired as the indicators of innovation. For example, Z. Zhang et al (2022) [3] stated that GCG improves the overall and incremental green innovations but impedes the radical green innovation of highly polluting enterprises with the framework of environmental policy, local government intervention and enterprise innovation.

In summary, most of the existing research results support the "Porter hypothesis", which also provides theoretical support for this study. GCG is a market-based regulation that reshapes the investment and financing mechanism of the environmental sector from the capital supply side outside the government sector. Its essence is an important extension and innovation of traditional environmental regulation. Enterprises that are more strongly affected by environmental regulations are more willing to carry out R&D innovation and have a larger amount of R&D expenditure. However, the implementation of a green credit policy will raise the financing threshold in the short term and directly aggravate the financing difficulty of enterprises to some extent. Therefore, compared with non-heavy polluting enterprises, when HPEs are faced with tighter credit regulation standards, they are more motivated to improve the quality of green development by improving innovation efficiency, to reach the new threshold of green credit.

## 3. Hypothesis development

The green credit policy has the dual functions of market capital allocation and environmental regulation, which binds the financial industry, environmental improvement and economic growth together. Banks, as creditors, bear a greater risk of investment failure than shareholders, while corporate management, in order to build a business empire, uses their free cash flow to invest in projects with net present value less than zero, thus infringing on the interests of creditors. Therefore, banks rationed credit to heavily polluting industries, adopted measures such as less loans and delayed loans to supervise the operation of borrowers, prevented financial and environmental risks, guaranteed their own credit security and improved credit quality. The introduction of GCG means that the financing environment faced by HPEs has changed greatly. Commercial banks incorporate environmental externalities into their credit decisions, conduct dynamic assessment and classification of enterprises’ environmental and social risks, provide credit investment to enterprises with lower energy consumption and emission levels, and form a dual constraint mechanism of environmental access and credit quota control for enterprises with high energy consumption and emission levels. When HPEs face external financing constraints, they may risk downsizing or even shutting down their business, which not only affects their operating profit but also causes higher sunk costs.

At this time, faced with tight green credit policy, it is not realistic for enterprises to carry out large-scale reseach and development (R&D) investment.Given limited R&D investment, we expect that enterprises will be more inclined to focus on the improvement of innovation efficiency. Therefore, compared with non-HPEs, when HPEs are faced with tighter credit regulation standards, they are more motivated to improve the quality of green development through improving innovation efficiency, so as to reach the new threshold of green credit.

The root cause of enterprise over-investment lies in the problem of principal-agent theory. According to the agency theory, due to the inconsistency between management and corporate shareholders, managers choose investment projects with net present value less than zero by using their free cash flow under the opportunistic motive, which leads to over-investment. Therefore, the management of companies with high free cash flow is more inclined to over-investment. Using free cash flow to make excessive investment is an important means for executives to obtain personal gain and also an important embodiment of management agency problem. However, excessive financing requirements pose a higher challenge to firms and can lead to reduced investment in innovation activities, effectively discouraging overinvestment. The less funds available to management, the less they can spend on investment, which leads to the limitation of management’s ability to overinvest opportunistically. Therefore, green credit policies can alleviate the agency problem of HPEs by reducing the discretionary cash flow of the managers, so as to restrain the over-investment behavior of heavy polluting enterprises.

With the gradual improvement of external incentives and constraints such as environmental information disclosure, promulgation of environmental laws and environmental regulations, HPEs will face strong external constraints on pollution emission, forcing them to assume social responsibilities for environmental protection, taking the initiative to carry out green transformation and innovation, and forming positive externalities of environmental protection. GCG internalize the positive externalities of environmental protection. By improving the availability of corporate credit and reducing financing costs (such as preferential interest rates), GCG alleviate the under-investment of HPEs and improve the investment efficiency of them.

Based on the above analysis, this paper proposes the following hypothesis:

H1a: GCG has a significant inhibitory effect on inefficient investment in innovation of HPEs.
H1b: GCG can effectively curb over-investment and under-investment of HPEs.

With different ownership properties, enterprises can bear different marginal costs. Therefore, enterprises need to weigh the rising cost of corporate debt financing in the operation process. At present, most of the credit resources of our financial institutions are captured by state-owned enterprises (SOEs), which causes private enterprises to face the common credit discrimination. When the GCG was promulgated, the heavily polluting private enterprises with narrow debt financing channels were unable to maintain a high sensitivity to the GCG, resulting in a lack of innovation motivation. However, before the implementation of the policy, the share of debt financing involved in state-owned HPEs is usually high, leading to the greater impact of GCG on them. Therefore, state-owned HPEs should be more motivated to innovate, improve their own level of green development, and respond to the dividend of green finance policy [40].

The degree of regional financial development will also affect the specific implementation of GCG. Under normal circumstances, GCG will raise the entry threshold of corporate loans. When a region’s financial system is more developed, companies have wider access to financing. In addition to credit financing, enterprises also have equity financing, bond financing and other financing options. Therefore, the regulatory role of GCG will be weakened in regions with developed financial industries. However, in areas with less developed financial development, enterprises are faced with severe financing environment and limited financing options. As the basic source of financing, enterprises are highly dependent on bank loans. Therefore, for HPEs located in less financially developed regions, GCG should provide stronger incentives for innovation.

Based on the above analysis, this paper proposes the following research hypotheses:

H2a: GCG has a more pronounced inhibitory effect on the inefficient investment innovative behavior of state-owned and less financially developed heavy polluters.

Another important issue in this paper is: How does GCG affect the efficiency of corporate investment? On the one hand, the sharp rise of environmental risks poses a threat to the safety of bank credit funds. According to the GCG, banks and other financial institutions take environmental risk factors as an important basis for credit and conduct strict credit assessment on HPEs, thus reducing credit investment in heavily polluting areas. In addition, GCG will further increase the transparency of enterprises’ disclosure of environmental information, which will in turn affect their financing ability in the capital market through the signaling effect [41]. As a result, HPEs face serious financing constraints. Long-term debt is the main source of funds for investment, and the impact of green credit on the maturity structure of debt will inevitably make management’s investment tend to be cautious and weaken the incentive of blind investment expansion. On the other hand, enterprises aim at profit maximization, and in order to alleviate the pressure of restricted debt financing and meet profitable production needs, they will actively seek alternative financing. At the same time, commercial credit has been found to be a common form of informal financing that can serve as an important alternative financing when firms face financing constraints. Therefore, despite the fact that green credit limits debt financing, it can avoid under-investment by firms due to financing constraints to some extent by increasing the input of commercial credit. In addition, commercial credit has a signaling effect, which helps to reduce the information asymmetry between banks and enterprises, thus forming effective supervision of enterprises and maximizing investment value.

In summary, this paper proposes the following hypotheses:

H3a: GCG reduces the proportion of long-term debt of heavy polluters, which in turn improves the investment efficiency of enterprises.
H3b: GCG increases the commercial credit size of HPEs, which in turn improves their investment efficiency.

## 4. Methodology and data

### 4.1 Data source

This study utilizes panel data of listed companies in Shanghai and Shenzhen A-shares from 2010 to 2020, and the following treatments are carried out: (1) exclude the samples of listed companies in the financial category; (2) exclude the samples with missing data of relevant indicators; (3) exclude the samples of ST, *ST and PT during the study period; (4) exclude the samples with asset-liability ratio greater than 1, and finally obtain 17,911 samples of 2,988 companies. Meanwhile, in order to enhance the robustness of the regression results, this paper conducts one-to-one matching according to the relevant variables in the relevant studies to alleviate the bias of the study results arising from the difference in sample self-selection in the two groups that are heavy polluting enterprises (treatment group) and non-HPEs (control group), and obtains 7599 data of 2611 companies after PSM matching. The data were obtained from the annual reports of enterprises, the CSMAR database. To eliminate the effect of extreme values, all continuous variables are winsorized by 1% in the regression.

## 4.2. Description of variables

### 4.2.1. Dependent variable

Our dependent variable is the inefficient innovation investment of HPEs. Inefficient innovation investment refers to the deviation of the actual level of innovation investment from the optimal level of innovation investment under realistic business conditions. Organizational factors are the main factors affecting innovation investment of enterprises.

Therefore, based on inefficiency investment model principle [42], and considering the impact of the sustainability of enterprise innovation investment and the number of enterprise patent applications in the current period, the following model (1) is constructed. Among them, the residual in model (1) refers to the level of innovation inefficiency investment, and the larger the absolute value of the residual, the higher the degree of innovation inefficiency investment. To further distinguish the types of innovation inefficiency investment, the samples with a residual value greater than 0 are defined as over-invested in innovation (over_Inn_Effi), and the samples with a residual value less than 0 are defined as under-invested in innovation (down_Inn_Effi). Considering that the residual value may contain the influence of missing variable factors, which leads to the deviation of the measurement of inefficient investment, samples with absolute residual value greater than 1% and 5% are selected for robustness test, respectively.


$$\ln{R\&D}_{it}=\ln{R\&D}_{it-1}+{Growth}_{it-1}+{Size}_{it-1}+{Lev}_{it-1}+{Cash}_{it-1}+{FirmAge}_{it-1}+{Ret}_{it-1}+{Pat}_{it}+{Year}_{t}+{Industry}_{i}+\varepsilon_{it}$$ (1)

#### 4.2.2. Independent variable

The implementation of Green Credit Guidelines is an important observation event in this study, we utilized DID(DID = treatment*post) as the main independent variable. Among which treatment is the dummy variable of a heavily polluting enterprise, which takes 1 when the company is in a heavy polluting industry, and 0 otherwise. Post is the dummy variable of the years before and after the implementation of GCG. If the company’s annual period is after 2012 (including the current year), it is 1; otherwise, it is 0.

#### 4.2.3. Control variable

According to the research process of [23–25],we control the company characteristic variables as shown in Table 1.

**Table 1:** Control variables.

Variable	Meaning	Measurement Index
Inn_Effi	Innovative inefficient investment	Absolute residual value
over_Inn_Effi	Over-investment in innovation	Sample residuals are greater than 0
down_Inn_Effi	Under-investment in innovation	Sample residuals are less than 0
Treatment	Group dummy variable	1 for heavy polluting enterprises and 0 for non-heavy polluting enterprises
Post	Event dummy variable	0 from 2010–2012 and 1 from 2012 to 2020
Size	The size of the corporate	The natural logarithm of the total assets at the end of the year
ROA	Net interest rate on total assets	Net profit/average balance of total assets
Lev	Asset-liability ratio	Total liabilities at year-end/total assets at year-end
Growth	Revenue growth rate	(Current Year’s operating revenue—last year’s operating revenue)/last year’s operating revenue
Dual	Two jobs in one	1if the president and the general manager are the same person;0 otherwise
Top1	Shareholding ratio of the largest shareholder	Number of shares held by the largest shareholder/ total number of shares
SOE	State ownership	1if state-owned;0 otherwise
FirmAge	Enterprise age	Ln (Year of the year—date of establishment +1)
TobinQ	Tobin’s Q	(Market value of tradable shares + market value of non-tradable shares * net asset value per share + book value of liabilities)/total assets
Year	annual	Time fixed effect
Industry	industry	Industry fixed effect

### 4.3. Research method

The PSM-DID method is widely used to study the effects of policies, DID method can effectively identify the different role effects of policies after their implementation, and it is the mainstream method in the current research on the role effects of policy class research. The key explanatory variables in Model (2) are green credit policies (Post), industry attributes (Treatment) and the cross-product term between them (DID). Policy is the dummy variable before and after the implementation of the Guidelines (2012). The value of this variable in the period after the implementation (2012 and later) is 1, and the value of it in the period before the implementation (before 2012) is 0. According to the Guidelines, the former China Banking Regulatory Commission specified the types of environmental and social risks in the Key Evaluation Indicators of the Implementation of Green Credit. In this paper, the industry of the enterprise with class A environmental and social risks were identified as whether the listed company was a green credit restricted industry. Specifically, Category A enterprises belong to 9 industries, including nuclear power generation, hydroelectric power generation, water conservancy and inland port engineering construction, coal mining and washing industry, oil and natural gas mining, black metal mining, non-ferrous metal mining, non-metallic mining, other mining industries. If the listed company belongs to the above 9 industries, it is identified as the green credit restricted industry (experimental group), Treatment = 1; otherwise, it is identified as the non-green credit restricted industry (control group), Treatment = 0. What we are most concerned about is the interaction term DID (Post × Treatment), which examines the impact of the implementation of the Guidelines on inefficient innovation investment in industries with and without green credit restrictions. If *β*_3_ is significantly lower than 0, it indicates that the Guidelines significantly deter inefficient innovation investment in the green credit-restricted industries; otherwise, it indicates that there is no significant inhibition effect.


$$Inn\_Effi=\beta_{0}+\beta_{1}Post+\beta_{2}Treatment+\beta_{3}DID+\beta_{i}{Controls}_{i}+Y_{t}+T_{i}+\varepsilon$$ (2)

According to the theoretical analysis, green credit policy inhibits the investment efficiency of heavy polluting enterprises by forming financing constraints. Based on this, this paper selects the SA index as the proxy variable of financial constraints for the empirical test conducted. Based on the mediation effect test method proposed, the following test equation is constructed on the basis of policy effect identification in the first step:

$$Over\;Inn\_E=\beta_{0}+\beta_{1}Post+\beta_{2}Treatment+\beta_{3}DID+\beta_{i}{Controls}_{i}+Y_{t}+T_{i}+\varepsilon$$ (3)

$${SA}_{i}=\alpha_{0}+\alpha_{1}Post+\alpha_{2}Treatment+\alpha_{3}DID+\alpha_{i}{Controls}_{t}Y_{t}+T_{i}+\varepsilon$$ (4)

$$OverInn\_E=\gamma_{0}+\gamma_{1}Post+\gamma_{2}Treatment+\gamma_{3}{SA}_{i}+\gamma_{4}DID+\gamma_{i}{Controls}_{t}{+Y}_{t}+T_{i}+\varepsilon$$ (5)

*β*_3_ significantly negative means green credit policy negatively affects the investment efficiency of heavily polluting enterprises, only when *β*_3_ significantly positive means green credit policy negatively affects the long-term credit financing situation of enterprises, α_3_ significantly positive means the next step test, in model (4), when γ_3_ and γ_4_ are significant can prove the intermediation effect between green credit policy and investment efficiency.


$$DownInn\_E=\beta_{0}+\beta_{1}Post+\beta_{2}Treatment+\beta_{3}DID+\beta_{i}{Controls}_{i}+Y_{t}+T_{i}+\varepsilon$$ (6)

$${TC}_{i}=\alpha_{0}+\alpha_{1}Post+\alpha_{2}Treatment+\alpha_{3}DID+\alpha_{i}{Controls}_{t}+Y_{t}+T_{i}+\varepsilon$$ (7)

$$DownInn_{E}=\gamma_{0}+\gamma_{1}Post+\gamma_{2}Treatment+\gamma_{3}{TC}_{i}+\gamma_{4}DID+\gamma_{i}{Controls}_{t}{+Y}_{t}+T_{i}+\varepsilon$$ (8)

## 5 Empirical results

### 5.1. Descriptive statistics

Table 2 is the descriptive statistics for the main variables. From the total sample, the mean of corporate inefficient investment of innovation (Inn_Effi) is 0.320, the median value is 0.189, the min and maxi are 0.000 and 17.190, and the standard deviation is 0.571. This indicates that most of the sample enterprises deviate from the optimal level of innovation investment, and there is a great variation among them. From the subsample results of over-investment in innovation (over Inn_Effi) and insufficient investment in innovation (down Inn_Effi), the means for them are 0.309 and 0.332, respectively, which shows that the proportion of insufficient investment in innovation of sample enterprises is relatively high and the differences in the latter is more obvious. In terms of the influence after the promulgation of the 2012 Guidelines (DID), the mean and median of green credit policy are 0.491 and 0.000, respectively, which indicates the sample enterprises are affected by green credit policy at a higher level than the medium development level. Other variables are within a reasonable value range. The results in Table 2 also show the mean of inefficient investment of innovation, over-investment in innovation and insufficient investment in innovation in the sample affected by GCG are significantly lower than those not affected by GCG, thus tentatively concluding that GCG helps to reduce the innovative inefficient investment behavior of firms and is beneficial to reduce over-investment in innovation and insufficient investment in innovation in HPEs.

**Table 2:** Descriptive results.

Total Sample							NDID		DID		T-test
Variable	N	Mean	SD	Min	P50	Max	N1	Mean1	N1	Mean2	
Inn Effi	7754	0.320	0.571	0.000	0.189	17.190	3946	0.348	3808	0.291	0.057***
over Inn E	3953	0.309	0.466	0.000	0.186	7.887	1932	0.338	2021	0.282	0.056***
down Inn E	3801	0.332	0.663	0.000	0.192	17.19	2014	0.358	1787	0.301	0.057***
DID	7754	0.491	0.500	0.000	0.000	1.000	——	——	——	——	——
Size	7754	22.360	1.287	19.080	22.150	28.480	3946	22.37	3808	22.34	0.026
ROA	7754	0.043	0.067	-0.662	0.039	0.880	3946	0.042	3808	0.045	-0.002
Lev	7754	0.412	0.197	0.008	0.403	1.345	3946	0.414	3808	0.409	0.005
Growth	7754	0.219	1.815	-0.862	0.097	84.990	3946	0.204	3808	0.236	-0.032
FirmAge	7754	2.905	0.316	1.386	2.944	3.970	3946	2.904	3808	2.906	-0.002
Dual	7754	0.254	0.435	0.000	0.000	1.000	3946	0.256	3808	0.252	0.004
Top1	7754	0.342	0.145	0.022	0.323	0.891	3946	0.342	3808	0.342	0.000
Dturn	7754	-0.032	0.391	-3.460	-0.003	2.062	3946	-0.033	3808	-0.032	-0.001
Indep	7754	0.373	0.054	0.231	0.333	0.800	3946	0.373	3808	0.373	0.001
TobinQ	7754	2.048	1.386	0.674	1.625	22.570	3946	2.034	3808	2.062	-0.027

### 5.2. Baseline results

According to model (2), Table 3 shows the regression results for the influence of GCG on the inefficient investment of innovation of HPEs. Column (1) reports the regression results for total inefficient investment, and columns (2) and (3) is the regression results for over-investment in innovation and under-investment in innovation. The coefficient of GCG (DID) in column (1) is significantly negative (β = -0.103, t = -6.02), indicating that GCG can effectively reduce inefficient innovation investments in sample enterprises.

**Table 3:** Baseline results.

	Inn_Effi	over_Inn_Effi	dowm_Inn_Effi
DID	-0.103***	-0.070***	-0.138***
	(-6.02)	(-3.65)	(-4.87)
Size	0.011	0.014*	0.009
	(1.58)	(1.89)	(0.77)
ROA	-0.302***	0.164	-0.678***
	(-2.81)	(1.39)	(-3.56)
Lev	0.183***	0.265***	0.119*
	(4.28)	(5.32)	(1.73)
Growth	0.044***	0.045***	-0.038
	(12.51)	(16.17)	(-1.43)
FirmAge	0.085***	0.065***	0.080***
	(3.72)	(2.56)	(2.11)
Dual	-0.030***	-0.035***	-0.031
	(-2.00)	(-2.11)	(-1.23)
Top1	-0.038	0.015	-0.098
	(-0.83)	(0.30)	(-1.27)
Dturn	-0.019	-0.012	-0.025
	(-1.02)	(-0.55)	(-0.84)
Indep	-0.092	0.012	-0.247
	(-0.78)	(0.09)	(-1.24)
TobinQ	0.018***	0.006	0.031***
	(3.40)	(1.05)	(3.14)
_cons	0.569***	0.024	1.213***
	(3.12)	(0.12)	(3.88)
Year	Yes	Yes	Yes
Industry	Yes	Yes	Yes
*N*	7754	3953	3801
adj. *R*^2^	0.067	0.133	0.050
F	14.584	16.168	5.995
p	0.000	0.000	0.000

The coefficient of DID in column (2) is negative and significant at the 1% level, with a value of 0.070. The results show that GCG can effectively mitigate 0.07% of innovation over-investment in HPEs. The coefficient of DID in columns (3) is significantly negative (β = -0.138, t = -4.87), which means that GCG can effectively mitigate 13.8% of innovation insufficient investment in HPEs.

### 5.3. Robustness test

#### 5.3.1. Placebo test

**Fig 1:**
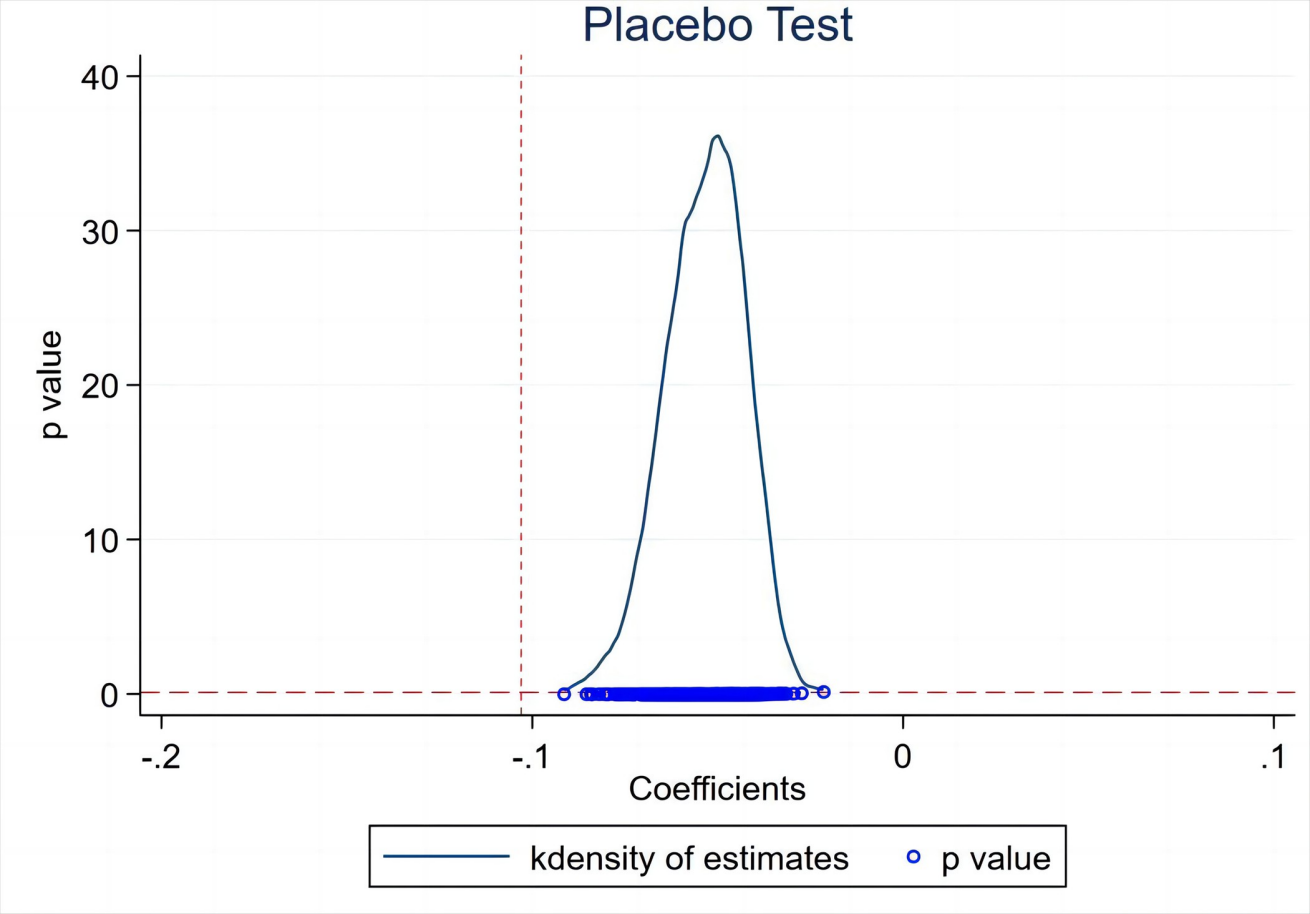
Placebo test.

It has been shown previously that green credit policies are effective in reducing innovation inefficiency investments in heavy polluters, but not in reducing unobservable systematic bias in the experimental and subject groups. To ensure the robustness of our findings, we conducted a placebo test on the sample enterprises. After randomizing the treatment group and sampling treatment group variables 500 times, we obtained kernel density curves for the coefficients of the randomized DID terms. To assess whether these coefficients are concentrated around 0 and significantly deviate from their true values, we randomly selected 50 cities as the treatment group and generated "pseudo-policy dummy variables" for 500 regressions. Subsequently, we plotted the distribution of coefficients and p-values for the 500 experimental results, as depicted in Fig 1. The blue dots represent p-values corresponding to the estimated coefficients, the vertical dashed line represents the true estimate of the DID model, and the horizontal dashed line indicates the 10% significance level. The red vertical dashed line denotes the test value for DID in the baseline regression (-0.103).

As can be seen in Fig 1, when Inn_Effi is the dependent variable, the coefficients of the model after 500 estimations are mostly distributed in the interval [-0.1 0.0]. The true estimated coefficients of the current study deviate far from this interval, which indicates that our estimated coefficients were not obtained by chance and proves the robustness of the above results.

#### 5.3.3. Impacts of other policies

This study studied the impact of GCG on inefficient investment of innovation of HPEs. However, during the study period, China also enacted many policies to alleviate corporate financing constraints, such as the Science and Technology Finance Policy (STFPP), the Twelfth Five-Year Plan (TFYP), and the Guidance on Green Financial System (GEGFS), which may lead to bias in the above findings. In order to eliminate the influence of these policies on this study and to verify the robustness of the above findings, the three policies mentioned above were added as control variables to the model (1). The final results are shown in Table 4, we can find that after excluding the effects of these three policies, the coefficient of DID is still significant at the 1% significance level, which proves the robustness of the above findings.

**Table 4:** Impacts of other policies.

	Inn_Effi	over_Inn_Effi	dowm_Inn_Effi
DID	-0.109***	-0.097***	-0.124***
	(-7.83)	(-5.63)	(-5.63)
STFPP	-0.281***	-0.228***	-0.343***
	(-5.66)	(-3.76)	(-4.37)
TFYP	-0.254***	-0.185***	-0.331***
	(-5.11)	(-3.04)	(-4.23)
GEGFS	-0.032	-0.043*	-0.022
	(-1.46)	(-1.68)	(-0.59)
Controls	Yes	Yes	Yes
Year	Yes	Yes	Yes
Industry	Yes	Yes	Yes
_cons	0.294***	-0.175	0.890***
	(2.03)	(-1.02)	(3.74)
*N*	11180	5722	5458
adj. *R*^2^	0.069	0.125	0.050

### 5.4. Heterogeneity test

To further explore the impact of the GCG on the inefficient investment of innovation of HPEs, we examine the ownership and financial development differences on whether this effect is more prominent in these diverse groups. The results are shown in Table 5.

**Table 5:** Heterogeneity test.

	SOE Inn_Effi	NSOE Inn_Effi	Developed Inn_Effi	Developing Inn_Effi
DID	-0.114***	-0.095***	-0.091***	-0.166***
	(-3.14)	(-5.27)	(-6.24)	(-3.97)
Year Industry	Yes	Yes	Yes	Year
	Yes	Yes	Yes	Industry
P-test N	0.085	0.960		
	2671	5083	9157	2023
adj. R2	0.061	0.084	0.075	0.050

#### 5.4.1. Heterogeneity of ownership

Columns (1) and (2) report the results of the ownership heterogeneity test. Considering the background of China’s institutional environment, there are some unequal competitions between state-owned enterprises (SOEs) and private enterprises (POEs). Since the government has stronger credibility as the actual controller of SOEs, SOEs have easier access to bank loans than POEs. Therefore, compared to POEs, the GCG is less constraining to the financing of heavily polluting SOEs, which can effectively alleviate the problem of insufficient investment in HPEs. It can be expected that the implementation of the Guidelines will have a more pronounced mitigation effect on the inefficient investment of innovation of state-owned HPEs. Based on the above analysis, this study introduces ownership variables for group testing to examine the inhibitory effect of GCG on inefficient investment of innovation of HPEs under different forms of ownership. The regression results in columns (1) and (2) of Table 5 show that GCG has a negative effect on the inefficient investment of innovation of HPEs. In addition, the absolute value of the coefficient of DID is larger in the sample of SOEs, (11.4% > 9.5%) which indicates that the GCG has a greater inhibitory effect on inefficient innovation in state-owned HPEs. This can also be further demonstrated by the component coefficient test (p = 0.085), which demonstrates a significant difference between groups.

#### 5.4.2. Heterogeneity of financial development

Columns (3) and (4) show the results of the heterogeneity test for the degree of regional financial development. We use the marketization index score of Wang et al to measure the degree of financial development in the region. We separate the sample enterprises into financially developed regions (Developed) and less financially developed regions (Developing) according to the median level of the score. The degree of financial development in a region can facilitate or limit the financing channels and options for local enterprises. Especially in less financially developed regions, enterprises face limited financing options and will be more influenced by GCG. The coefficients of DID are -0.091 and -0.166, respectively, and both are significant above the 1% level. This indicates once again that the GCG has a significant mitigation impact on the innovation investment efficiency of HPEs. By comparing the absolute values of the coefficients of DID, the GCG has a more significant inhibitory effect on inefficient investment of innovation of HPEs in the less financially developed regions.

### 5.5. Mechanism test

#### 5.5.1. Test for financing effects

Table 6 shows the results of the regressions on models (2)-(7). Column2 of Table 6 shows that the coefficient of DID is -0.097, which is significant at the 1% level, indicating that the GCG has a significant inhibitory effect on the inefficient investment of innovation of HPEs; column 3 of Table 6 shows that the coefficient of DID is 0.011, which is also significant at the 1% level, indicating that GCG increases the SA index, which means that the GCG increases the HPEs’ financing constraints and increased financing costs, which in turn inhibits overinvestment behavior in the innovation process of enterprises. Thus, the SA index financing condition indicator passes the mediating effect model test and the hypothesis that GCG affects overinvestment in HPEs and thus inefficient investment of innovation through financing constraints on HPEs is verified.

**Table 6:** Mechanism test.

	over_Inn_Effi	SA	over_Inn_Effi	down_Inn_Effi	credit	down_Inn_Effi
DID2	-0.097***	0.011***	-0.093***	-0.124***	0.190***	-0.108***
	(-5.63)	(13.83)	(-5.41)	(-5.63)	(16.54)	(-4.87)
Controls	Yes	Yes	Yes	Yes	Yes	Yes
Year	Yes	Yes	Yes	Yes	Yes	Yes
Industry	Yes	Yes	Yes	Yes	Yes	Yes
SA/Credit			-0.209*** (-3.61)			-0.090*** (-4.86)
_cons	-0.175	3.011***	0.438*	0.890***	2.153***	1.097***
	(-1.02)	(100.77)	(1.82)	(3.74)	(18.13)	(4.55)
*N*	5722	11180	5722	5458	11180	5458
adj. *R*^2^	0.125	0.805	0.127	0.050	0.211	0.054

#### 5.5.2. Test for commercial credit

Although the GCG restricts debt financing, it is able to avoid the lack of innovation investment by HPEs due to financing constraints by increasing the input of funds through commercial credit. Drawing on other literature, we adopts the ratio of the sum of accounts receivable and notes receivable to total assets to measure the commercial credit (TC) of enterprises, and divides them into high and low commercial credit groups based on the median of commercial credit values; those with TC values above the median are classified as high credit groups (HC), otherwise they are classified as low credit groups (LC).

Column (2) of Table 6 shows that the coefficients of DID are -0.124, -0.190 and -0.108, respectively, which are all significant at the 1% level, indicating that GCG improves the business credit of HPEs and the hypothesis that GCG inhibits the insufficient investment behavior of firms in the innovation process through business credit is verified.

## 6. Conclusion and policy implication

### 6.1. Conclusion

In recent years, economic development and ecological protection have received more attention from institutions and the general public. As an important financial control instrument to stimulate the green development of enterprises, green financial policies are gradually influencing the business decisions and innovative behaviors of relevant enterprises. This paper conducts a quasi-natural experiment to examine the effect of GCG on the inefficient investment in innovation of HPEs. The findings show that GCG can deter the inefficient investment of innovation of HPEs. And this result remains robust after a placebo test and a robustness test that excludes other policy disturbances. This result supports the existence of Porter’s hypothesis because our result proves that GCG can effectively reduce firms’ inefficient investment in innovation, which can enhance the effectiveness of innovation funding to stimulate firms’ innovation, thus effectively supporting that Porter’s hypothesis holds. The more firms are influenced by green credit policy, the less the scale of firms’ innovation investment deviates from the optimal investment level. Moreover, this effect is more prominent in state-owned HPEs and HPEs in less financially developed regions. As for the mechanism, GCG inhibit enterprise inefficient investment in innovation through financial constraints and business credit to ease overinvestment and underinvestment, respectively.

### 6.2. Discussion

This paper analyzes the impact of green credit policy on the inefficient investment in green innovation of heavy polluters, and analyzes the mechanism of action and the heterogeneous effect of action. The findings show that GCG can deter the inefficient investment of innovation of HPEs. And this result remains robust after a placebo test and a robustness test that excludes other policy disturbances. The more firms are influenced by green credit policy, the less the scale of firms’ innovation investment deviates from the optimal investment level. Moreover, this effect is more prominent in state-owned HPEs and HPEs in less financially developed regions. As for the mechanism, GCG inhibit enterprise inefficient investment in innovation through financial constraints and business credit to ease overinvestment and underinvestment, respectively. This paper has some limitations. Since we do not have access to firm-level or regional-level data on green credit in China, we only consider the impact of the 2012 Guidance after its promulgation, and not the results of its implementation in practice. However, the results achieved by GCP may be different from those expected. Therefore, a follow-up study on the implementation of GCP is needed. In addition, although green credit is the core of green finance, further research should also consider the role of other green finance policies on corporate environmental governance, such as green securities, green insurance, and green bonds.

### 6.3. Policy implications

The above study has policy implications for the government, financial institutions and heavy polluters. For the government, it should promulgate and implement more policy acts like green credit guidelines to promote corporate, social and environmental sustainability. In addition, when implementing policies, the government should also take regional differences into account and introduce programs that are suitable for each region according to local conditions to avoid a one-size-fits-all approach that may affect the effectiveness of the policy. For financial institutions like banks, they ‘d better adjust credit constraints and commercial credit loan conditions, especially for the transformation of heavily polluting enterprises can be appropriately relaxed conditions to promote the transformation and upgrading of enterprises and green development. In addition, banks also need to consider the degree of regional financial development to make appropriate adjustments. For the heavy polluters, relevant enterprises should improve production processes, promote green production, actively comply with laws and regulations and regulatory policies, improve corporate information disclosure mechanisms, and actively accept investor and government supervision.
